# Diphtheria

**Published:** 1899-10-21

**Authors:** P. Watson Williams

**Affiliations:** Physician to Clifton College; and Physician in Charge of the Department for Diseases of the Throat, Bristol Royal Infirmary.


### Hospital Clinics and Medical Progress.

### DIPHTHERIA.

By P. Watson Williams, M.D.Lond., Physician to Clifton College; and Physician in Charge of the Department for Diseases of the Throat, Bristol Royal Infirmary.

(*Continued from page* 28.)

**Symptoms.**—*Prodromal.* Diphtheria being primarily a local infection the initial symptoms are essentially local. In a few cases a sense of lassitude, aching in the limbs and back, and chilliness, accompanied by some rise of temperature and general febrile disturbance, precede the throat symptoms by a few hours, or, rarely, even a few days.

**Local Symptoms** in the fauces, consisting in slight or considerable soreness, especially on swallowing, cough, and other symptoms of acute pharyngitis, and pains in the neck, are usually the first indications of the onset of an attack of pharyngeal diphtheria. If the fauces are examined at this early stage they will be found hyperæmic, swollen, and glazed, while the glands at the angles of the lower jaw may have begun to swell.

After an interval varying between a few hours and a few days, a false membrane generally, though not always, begins to form one or more patches on the tonsils and uvula, or it may begin in small specks on the margins of crypts of the tonsil or on the uvula, spreading more or less rapidly, till by their coalescence these small patches, at first thin and translucent, become thicker and unite to form a characteristic tough, opaque, greyish-white false membrane. This may completely cover the uvula, soft palate, and tonsils, and extend to the pharyngeal walls. Sometimes the patches remain discrete, so as to closely resemble acute follicular tonsillitis, punctiform diphtheria. The small white patches are on the surfaces of the tonsil, not in the crypts, and cannot be squeezed out. Moreover, some will generally be found to present a rugged edge, unlike the accumulation within a tonsillar crypt. Yet other cases are precisely similar in appearance to simple acute lacunar tonsillitis, and it would baffle the acutest diagnostician to differentiate the one from the other without a bacteriological examination.

Marked rapidity in formation and the extent of the false membrane is some indication of the virulence of the disease, but is no reliable criterion. The pellicle is generally firmly adherent to the underlying mucosa, and on its removal a raw, ragged, and bleeding surface is formed.

In some cases no pellicle is formed at any period in the attack, and these are by no means always mild. In others the membrane, instead of being thick, tough, and adherent, is thin or friable, flocculent, and simply lying free, or so lightly attached to the surface of the mucous membrane that it can be readily removed by lightly brushing, leaving a slightly abraded or almost normal surface. Again, instead of being white or greyish-white in colour, the membrane may be a dark ash-grey or blackish or greenish-grey, and copious muco purulent discharge, with a foul odour, collects in the throat, the so-called putrid sore throat of older writers.

Speaking generally, the firm, whitish-grey membranes give almost pure cultures of the diphtheria bacillus, while the dark grey, yellowish, or black, and the flocculent friable membranes yield mixed cultures of the specific bacillus together with various pyococci and saprophytic micro-organisms. Opinions are divided as to the influence of mixed growths in the course of the attack; thus Martin, Funk, Roux, and most of the French school consider that the association of streptococci or staphylococci exerts a most unfavourable influence, but Washbourn, Kanthack, and others are of opinion that their presence does not in itself render the prognosis more unfavourable. I am inclined to regard a mixed culture, showing copious growth of pyococci (on a medium unsuited to their growth and examined within 24 hours) as distinctly unfavourable, for, though there are scarcely any cases in which prolonged culture and careful examination do not show their presence at any rate in small numbers, most hæmorrhagic and septic complications of diphtheria, such as suppurative adenitis, suppurative inflammation in the middle ear, and septicæmia, are due to the action of these pyococci, which diphtherial antitoxin cannot control. It probably depends on the initial virulence of the 'cocci whether they exert a pernicious influence, and increase the tendency to septic complications.

The lymphatic glands at the angles of the jaws and in the neck become swollen and tender, but rarely suppurate. In malignant cases the swelling is considerable, and extends beyond the glands, so that the whole of the neck from the chin to the upper part of the chest may be enormously swollen. The membranous inflammation may extend to the larynx, and down the trachea and bronchi, or upwards to the nose, giving rise to special features.

In rare cases false membranes have been found in the stomach and on the Peyer's patches in the intestines.

**General Symptoms.**—At the onset the temperature generally rises, but seldom exceeds 103 deg. Fahr. or 104 deg. Fahr., often falling slightly on the second or third day, and continuing moderately febrile for a few days longer. In very mild cases and in the severer forms the temperature is generally lower, and may be nearly normal, or in the worst cases subnormal, owing to the depressing influence of the poison in the circulation. The pulse, at first increased in frequency and of the usual febrile type, tends to become weak and small, especially in severe cases. The heart's action is correspondingly impaired, and early heart failure is apt to occur in malignant diphtheria, the heart sounds becoming more or less indistinct or inaudible. The onset of symptoms of cardiac weakness may be gradual or may set in suddenly, and in the worst cases sudden death may occur during the first few days, or the patient may gradually sink and become unconscious after remaining several days in a more or less algid state. Primary heart failure is most likely to occur between the fifth and the fourteenth day, but may arise many days later. The pathological changes in the cardiac muscle and nerves which result in cardiac weakness or failure have already been described.

The general symptom never absent, though varying greatly in degree, is prostration of strength. This feature may not be obvious within the first few days; on the other hand, it may develop so rapidly, and in such marked degree, that the patient may succumb to the depressing effect of the poison on the nervous system before the false membrane has had time to form. Rapid and pronounced anæmia and leucocytosis is usually observable, and in bad or fatal cases hæmorrhages from the mucous membranes, and general cyanotic tint of the lips and surface of the body may appear, apart from dyspnœa. Various rashes of short duration occur in diphtheria, *e.g.*, large patches of erythema resembling scarlatina, or a vesicular and urticarial rash may occur; these are without significance, but ecchymosis, indicative of septic poisoning, are of grave import. The tongue is furred, and the appetite lost. The spleen is usually enlarged.

The urine frequently contains albumin, which is very variable in amount. Much less often acute nephritis manifests itself by the presence of blood and casts in the urine. Various other complications and sequelæ may arise, apart from extension of the disease to the larynx or nose, &c., viz., lobular pneumonia, suppuration in the glands of the neck, and in the middle ear, septicæmia, and post-diphtheritic paralyses.

